# Two Faces of the Screened Bottom Boards—An Ambiguous Influence on the Honey Bee Winter Colony Loss Rate

**DOI:** 10.3390/insects13121128

**Published:** 2022-12-07

**Authors:** Ewa Danuta Mazur, Michał Czopowicz, Anna Maria Gajda

**Affiliations:** 1 Department of Pathology and Veterinary Diagnostics, Institute of Veterinary Medicine, Warsaw University of Life Sciences—SGGW, Nowoursynowska St. 159c, 02-776 Warsaw, Poland; 2 Division of Veterinary Epidemiology and Economics, Institute of Veterinary Medicine, Warsaw University of Life Sciences—SGGW, Nowoursynowska St. 159c, 02-776 Warsaw, Poland

**Keywords:** *Apis mellifera*, colony losses, overwintering, screened bottom boards, beekeeping

## Abstract

**Simple Summary:**

Polish beekeepers struggle with high winter colony losses almost every year. We investigated the effect of using screened bottom boards on the overall colony loss rate. We conducted a citizen science survey on the winter colony losses in Poland from 2017/18 to 2019/20. Beekeepers answered questions regarding the number of overwintered and lost colonies and basic beekeeping practices. The study shows that the use of screened bottom boards was associated with the reduced overall colony loss rate; however, the relationship with various types of colony losses was complex and multidirectional. Nevertheless, our study shows that the benefits outweigh the risks, and we recommend the use of screened bottom boards in beekeeping practice in Poland.

**Abstract:**

We conducted a citizen science survey on the winter honey bee colony losses in Poland from 2017/18 to 2019/20 to determine the influence of the use of screened bottom boards on the winter colony losses due to various causes. A total of 1035 beekeepers with 40,003 colonies reported valid data. The overall winter colony loss rate ranged from 10.7% to 13.9%, and in every year, the overall winter colony loss rate was higher than 10% (which is considered as acceptable in Poland). The study reveals that the use of screened bottom boards was associated with reduced overall loss rate. However, the nature of this relationship was not the same in terms of all types of colony losses: while the use of screened bottom boards was associated with a reduced mortality rate (management-related colony loss rate due to dead colonies) in which the empty hives were observed (colony depopulation syndrome, CDS), it was associated with an increased mortality rate in which the lack of food was observed (starvation). Given that in our study the role of CDS in the overall colony loss rate was 2.5-fold higher than the role of starvation, the final influence of the use of screened bottom boards on the overall colony loss rate turned out to be beneficial. Given the well-known beneficial role of screened bottom boards in varroosis control, they are highly recommended in beekeeping practices in Poland.

## 1. Introduction

Since 2008, Poland has been a part of the international honey bee winter loss monitoring program run by the Prevention of Honey Bee Colony Losses Association (COLOSS). COLOSS is an international non-profit association working to improve the well-being of bees at the global level. Its main objective is to mitigate the increased mortality of honey bee colonies. The first reports of increased losses came from the USA [1], and soon were followed by reports from Europe and other continents [2,3,4,5]. Honey bees play a crucial role as plant pollinators, and their increased mortality has a considerable negative impact on the global economy through the reduction in agricultural production and beekeeping production, mainly honey and wax, which are important trade products [6,7]. Further intensive studies on honey bee colony losses are highly warranted, especially in the face of new threats associated with changing climate [8]. 

It has become clear that in order to understand the losses in a global scale, the data collected in different countries must be comparable. To achieve this goal, the COLOSS monitoring core project group surveys beekeepers in their respective countries about their bee colonies. The survey is conducted annually and is based on a self-completed standardized questionnaire, which is formatted similarly in all participating countries. As the greatest honey bee colony losses occur during the overwintering period, the survey begins immediately after overwintering has been completed (in Poland this is usually in March), when beekeepers can visually inspect their colonies. The data collected from each country are coded by the national coordinator and protected according to the General Data Protection Regulation (GDPR). 

**Figure 1:**
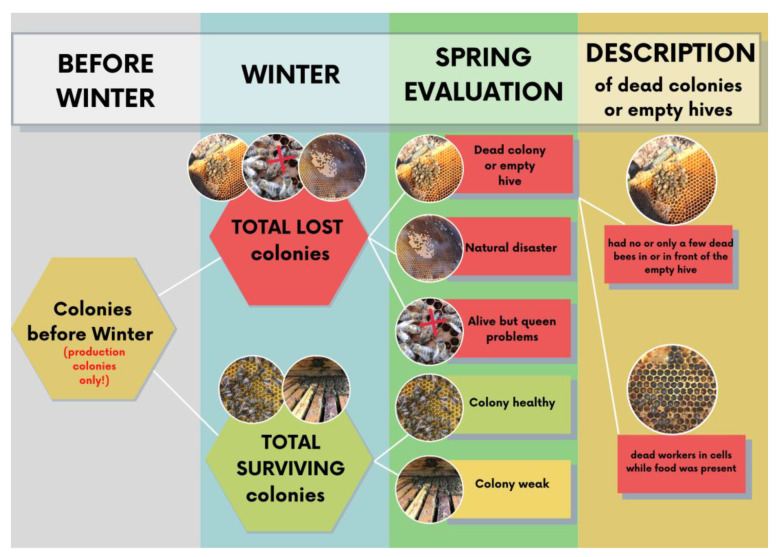
Possible causes of colony loss included in the COLOSS questionnaire. The diagram is based on the graphic created by Mariia Fedoriak, and is provided by courtesy of her.

Three basic categories of possible causes of winter colony losses are investigated in the COLOSS questionnaire: (1) unsolvable queen problem (i.e., death of queen or the presence of drone-laying queen); (2) natural disaster (flood, vandalism, etc.); and (3) dead colony (Figure 1). The latter is further subdivided, based on the character of the phenomenon that has led to colony death, into a dead colony with a handful of bees in the hive or in front of the hive (colony depopulation syndrome, CDS), and dead workers in cells without food on the frames (referred to as starvation). These symptoms may result from *Varroa destructor* infestation complicated by viral co-infections (i.e., deformed wing virus, DWV, acute bee paralysis virus, ABPV), *Nosema* spp. infection [9,10], or from the shortage of food. Considerable bee colony losses due to *V. destructor* infestation or *Nosema* spp. infection have been confirmed by studies from many countries [11,12,13,14,15]. There are other possible causes of colony death during winter, such as American foulbrood or dysentery caused by honeydew honey in winter food; however, these usually account for only single cases. 

Analysis of international data from the COLOSS questionnaire has already indicated several beekeeping practices with an important impact on colony survival. Of these, the number of colonies possessed by a beekeeper and the percentage of queens replaced during the season are linked to a reduced winter colony loss [16,17,18,19,20], while practicing migration of colonies appears to have an ambiguous influence on losses—in some studies it turned out to increase [17], in others to reduce losses [18,19]. 

An important element of the box multistory hive (“Wielkopolski” or “Dadant” in Poland) is the bottom board, which may be either solid or screened. The bottom board is the floor of the hive, and includes an entrance to the hive and a bottom board landing. The screened bottom board has a mesh instead of a solid bottom. It often has a removable bottom drawer or even the mesh itself may be mobile. The screened bottom board has undeniable advantages. It allows for quicker and more effective cleaning of the hive because it can easily be changed at any time, even when an active colony is inside. In addition, it facilitates the monitoring of various infectious and parasitic diseases, including varroosis [21,22]. On the other hand, the screened bottom board causes more intensive air circulation and ventilation inside the hive. Therefore, when it is cold outside, bees need to use up more energy to warm up the colony, which may lead to excessive food consumption. 

Screened and solid bottom boards may be used interchangeably—the former during warmer months and the latter as part of the overwintering preparation. However, it is common practice in the temperate climate regions to uninstall screened bottom boards (by inserting a bottom drawer) in mid-winter (January/February), because at this time, the queen begins to lay eggs and workers begin to warm the brood. Poland, located in central Europe, has a moderate climate with both maritime and continental elements. Over the last decade, the average winter temperatures have considerably increased in Poland [23], which may justify maintaining screened bottom boards even for the entire year. The impact of such a practice on the overall winter colony losses is, however, unknown. Therefore, we carried out the present study to determine an influence of the use of screened bottom boards for the entire winter on the winter colony loss rate due to various causes, taking into account other factors known to affect winter colony losses.

## 2. Materials and Methods

### 2.1. Questionnaire Survey

This was an analytical cross-sectional study based on the self-completed standardized COLOSS questionnaire (Figure S1). It was carried out in three consecutive seasons, 2017/18, 2018/19, and 2019/20, on the target population of Polish beekeepers. Every year, the questionnaire is launched immediately after the overwintering period, and remains available on the Internet platform (“Limesurvey”) for at least 3 months (from April to June). Information about the questionnaire with an access link is disseminated via three popular beekeeping magazines, social media, websites, conferences, lectures, and personal contact, to encourage beekeepers to participate in the study. Each year, e-mail invitations are also sent to beekeepers whose e-mail addresses are available in the database. To enable beekeepers without access to the Internet to join the study, a paper version of the questionnaire is published in one of the aforementioned magazines. An invitation to complete the questionnaire is also posted on the website of the Warsaw University of Life Sciences–SGGW as well as on various beekeeper organization’s websites. Moreover, in the season 2017/18 the paper questionnaire was also sent to beekeepers by post. The list of registered Polish beekeepers with their addresses was obtained from the Polish Veterinary Inspection with compliance to the GDPR.

The winter was defined as the period between the moment of finishing the pre-winter preparations (usually October) and the start of the new foraging season. The following data were extracted from the COLOSS questionnaire and analyzed in this study: (1) province of Poland in which a beekeeper located their beehives (answer to be chosen from 16 options); (2) the number of production colonies (with healthy queen and strong enough to provide honey harvest) owned by a beekeeper before the winter (numerical answer); (3) the number of colonies lost after winter (numerical answer); (4) the number of queens mated in the previous season (numerical answer); (5) migration of colonies for honey production or pollination services (dichotomous answer—YES/NO); (6) use of screened bottom boards in winter (dichotomous answer—YES/NO); (7) monitoring of *V. destructor* (dichotomous answer—YES/NO); (8) treatment against *V. destructor* (dichotomous answer—YES/NO).

The overall colony loss rate was calculated as the number of lost colonies after winter divided by the number of all production colonies before winter. The colony loss was classified by respondents into losses due to natural disasters, which are independent of the management, and management-related losses, which could be characterized by the presence of a living colony but with an unsolvable queen problem (e.g., death of the queen or drone-laying queen) or the presence of a dead colony (mortality). The latter category could optionally be further subclassified according to what beekeepers found in the hives with dead colonies into 2 options (in the standard version—5): empty hive without bees or only a handful of bees (CDS) or dead bees present in cells and no food on the frames (starvation). 

Beekeepers were also asked to provide the number of colonies with young queens (queens mated in the previous season). These numbers could not have been higher than the number of overwintered colonies, and were used to cross-verify the information regarding the total number of production colonies before winter.

### 2.2. Statistical Analysis

The database was created in the Microsoft Excel (Microsoft^®^ Excel^®^ 2019 MSO). Before analyses, the database was checked for doubling and incomplete or illogical answers or gaps. Only complete datasets were used, as this was required in the multivariable analysis. All colonies owned by one beekeeper (operation) were treated as one apiary. Categorical variables were presented as counts and percentages. Numerical variables were presented as the median, interquartile range (IQR), and range. The change in management practices in subsequent years was analyzed using the χ^2^ test for trends [24] or the Kruskal–Wallis H test, in the case of categorical and numerical variables, respectively. The winter colony loss was expressed as the overall colony loss rate, calculated by dividing the number of colonies lost due to a particular reason by the total number of colonies going into winter [25]. The mixed-effect binary logistic regression model was developed [25] to investigate the influence of the use of screened bottom boards on the following types of winter colony loss rates: overall winter colony loss rate, winter colony loss rate due to natural disasters, management-related factors, unsolvable queen problems, dead colonies (mortality), empty hives (CDS), and lack of food (starvation) [25]. In the univariable analysis, the models included the use of screened bottom boards fitted as a fixed effect and the year of the study fitted as a random effect. If the use of screened bottom boards turned out to be significantly linked to a particular type of winter colony loss in the univariable analysis, the multivariable mixed-effect binary logistic regression was conducted. This included a year of the study and a region in which the apiary was located fitted as random effects, and all management-related variables whose influence on colony loss rate had been proven in previous studies, and that could, therefore, produce a spurious association between study variables or mask a real association (confounders), fitted as fixed effects. These potential confounders were as follows: the number of colonies owned by a beekeeper and the percentage of queens replaced entered into the model as numerical variables; and the migration of colonies, varroosis monitoring, and varroosis treatment entered into the model as dichotomous variables. The strength of the relationship was expressed using the odds ratio (OR), specifically, crude OR (OR_crude_) in the univariable analysis and adjusted OR (OR_adj_) in the multivariable analysis. All statistical tests were two-tailed. The significance level (α) was set at 0.05. Statistical analysis was performed in TIBCO Statistica 13.3 (TIBCO Software Inc., Palo Alto, CA, USA) and IBM SPSS Statistics 28 (IBM Corporation, Armonk, NY, USA).

## 3. Results

In total, 1035 beekeepers provided data about 40,003 overwintering colonies. The number of respondents increased each year, as did the total number of colonies before winter (Table 1).

**Figure 2:**
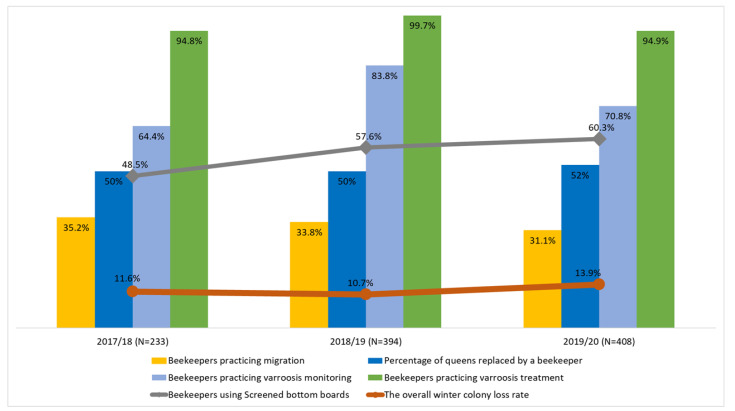
The overall winter loss rate and the beekeeping practices of Polish beekeepers surveyed in years 2017–2020.

The number of respondents who used screened bottom boards significantly increased over the 3 years from 48.5% to 60.3% (*p* = 0.008). The use of other beekeeper practices fluctuated in time, but without any significant trend. Respondents changed roughly 50% of queens in the hives each year (Figure 2). 

The overall winter colony loss rate was above 10% in all years (Figure 2). Natural disasters accounted for only a small proportion of all winter colony losses. Of the management-related winter colony losses, the dead colonies (mortality) accounted for roughly twice as many losses as the unsolvable queen problems. Of the dead colonies, the empty hives (CDS) were observed 2–3 times more often than the lack of food (starvation) (Table 2).

In the univariable analysis, the use of screened bottom boards was significantly linked with the overall winter colony loss rate as well as other types of winter colony losses, except colony losses due to natural disasters and due to unsolvable queen problems (Table 2). The nature of this relationship, however, was not the same in terms of all types of colony losses: while the use of screened bottom boards was associated with a reduced mortality rate (management-related colony loss rate due to dead colonies) in which the empty hives were observed (CDS), it was associated with an increased mortality rate in which the lack of food was observed (starvation). Since, in our study, the role of CDS in the overall colony loss rate was 2.5-fold higher than the role of starvation, the final influence of the use of screened bottom boards on the overall colony loss rate turned out to be beneficial. These results also held in the multivariable analysis (Table 2; Table S1–S5); however, the relationship between increased mortality rate due to starvation was even stronger when the influence of other management factors was concomitantly included (OR_adj_ = 1.69 compared to OR_crude_ = 1.49) (Table S5). As a consequence, this weakened the beneficial influence of the use of screened bottom boards on the management-related (OR_adj_ = 0.91 compared to OR_crude_ = 0.84) (Table S2) and overall winter colony loss rate (OR_adj_ = 0.91 compared to OR_crude_ = 0.85) (Table S1). In all analyses, the region of Poland in which the apiary was located was significantly associated with winter colony losses (*p* = 0.001).

## 4. Discussion

Our study shows that using screened bottom boards has a significant influence on the winter colony loss rates. This relationship is, however, complex and multidirectional. While screened bottom boards seemed to reduce mortality rate (management-related colony loss rate due to dead colonies) in which the empty hives were observed (CDS), they appeared to increase the mortality rate in which the lack of food was observed (starvation). Since, in our study, the role of CDS in the overall colony loss rate was higher (3.7%) than the role of starvation (1.4%), the final influence of the use of screened bottom boards on the overall colony loss rate was negative, i.e., when screened bottom boards were used, the overall colony loss rate was lower by roughly 2%. However, if the higher proportion of the overall colony loss rate was associated with the lack of food more than with empty hives, the benefits from using screened bottom boards would vanish, or could be even replaced by losses.

The screened bottom boards are considered useful in managing bee colonies, especially in terms of disease prevention and control. The screened bottom boards have proven efficient in varroosis infestation monitoring [21,26], as well as in reducing mite populations, for which they were actually designed [27]. A positive effect of screened bottom boards on colony loss rates in the USA only occurred when other non-chemical varroosis treatments were used [28]. These results are consistent with a study from Pakistan [29], where varroosis control using oxalic acid and screened bottom boards was found to be very effective. Some studies have also proven the relationship between the use of screened bottom boards and lower varroosis prevalence [30,31]. The highest colony losses in Poland are associated with symptoms of CDS [5], which are linked to high *V. destructor* infestation and virus or *Nosema* spp. infection. The main advantage associated with the use of screened bottom boards is the ability to monitor varroosis infestation as well as the effectiveness of treatment. Checking the mite fall on the bottom boards is one of the main monitoring options chosen by beekeepers in Poland (unpublished data). In addition, one of the most popular treatments for varroosis is fumigation with amitraz (Apiwarol^®^, Biowet Puławy, Puławy, Poland), and the mesh bottom board is useful in its application. Moreover, hive debris is a valuable sample source for detecting bee diseases [22,32,33]. 

Given that the beekeeping sector has to adapt to climate change [8], another advantage of screened bottom boards is their positive impact on hive ventilation in the summer and/or autumn. Warm autumns have been shown to result in high winter losses in Poland [34]. However, our study shows that in colonies with symptoms of starvation, the use of screened bottom boards was associated with significantly higher overall loss rate. Presumably this is due to increased ventilation and temperature fluctuations during the winter. A natural bee nest with thick walls creates the insulation that helps bees maintain the optimal temperature of the colony. Hives with thin walls and screened bottom boards do not ensure adequate insulation, so the bees need more energy (more food) to survive [35]. The higher ventilation in the hive (due to the mesh bottoms) has been shown to negatively affect bees during overwintering [36]. It is possible that the effect of screened bottom boards also depends on the location of the apiary in Poland. It is likely that in the coldest regions of Poland (north-east [23]), screened bottom boards have a negative effect on the overwintering of colonies. It should be emphasized that the lack of food may at least partly be related to the beekeepers’ mistake consisting in providing bees with an insufficient amount of food [35]. On the other hand, a study from a subtropical climate showed that the positive effect of using screened bottom boards during the winter was observed in colder locations, when, despite intensive sunshine, bees kept on forming tight clusters. Due to screened bottom boards, the temperature inside the hive was low enough to prevent bees from flying, which resulted in reduced colony losses [37]. Given the increasingly warmer autumns and winters in Poland, the use of screened bottom boards is advisable in order to lower the temperature inside the hives. 

In this study, we did not investigate the role of the potential interaction between the use of screened bottom boards and a particular varroosis treatment method, which may be significant. We included varroosis monitoring and varroosis treatment as surrogates of the high prevalence of this parasitic disease in Poland. It is known that *V. destructor* is one of the most serious global threats to honey bees [38], and it is widespread in Poland [39]. Other confounders, such as the number of colonies owned, migration, and queen replacement, were found to be significantly linked to bee colony mortality (Tables S1–S5), as has been shown in previous studies from other countries [14,17,19]. 

## 5. Conclusions

Our study shows that the popularity of using screened bottom boards during overwintering increased over the study years. However, their influence on the overall loss rate is complex. Given the benefits of screened bottom boards in varroosis control, they are highly recommended in beekeeping practices in Poland. However, beekeepers should remember to prepare enough food storage for the colonies, and monitor the thermoregulation and ventilation of the colonies. Further investigations are warranted to gain insight into the influence of the type of bottom board on colony mortality in regions with different environmental and climatic conditions, as well as the effect of screened bottom boards on the development and productiveness of bee colonies during different seasons. 

## Figures and Tables

**Table 1:** The general characteristics of the study population of Polish beekeepers surveyed in years 2017–2020. N = number of respondents.

	Overall	2017/18 (N = 233)	2018/19 (N = 394)	2019/20 (N = 408)
No. of colonies owned by a beekeeper ^a^	23, 12–43 (1–800)	25, 12–47 (1–498)	25, 13–44 (1–500)	21, 11–38 (2–800)
The total number of colonies before winter	40,003	9350	15,023	15,630
The total number of colonies lost after winter	4867	1085	1611	2171
The overall winter colony loss rate (95% confidence interval, CI 95%)	12.1 (10.3–14.2)	11.6 (9.7–13.9)	10.7 (9.2–12.4)	13.9 (12.2–15.7)

^a^ Presented as the median, interquartile range (IQR), and range.

**Table 2:** Univariable analysis of the relationship between the use of screened bottom boards and the winter colony loss rate.

The Winter Colony Loss Rate (95% Confidence Interval, CI 95%):	Entire Study Population (n = 1035)	Beekeepers Using		Univariable Analysis ^a^		Multivariable Analysis ^b^	
		Screened Bottom Boards (n = 586)	Solid Bottom Boards (n = 449)	*p*-Value	Crude Odds Ratio (OR_crude_) (CI 95%)	*p*-Value	Adjusted Odds Ratio (OR_adj_) (CI 95%)
**Overall**	12.1 (10.3–14.2)	11.3 (9.5–13.3)	13.0 (11.0–15.3)	<0.001	0.851 (0.801–0.905)	0.003	0.905 (0.847–0.967)
Due to:							
Natural disaster	0.4 (0.3–0.5)	0.4 (0.3–0.6)	0.4 (0.3–0.6)	0.538	1.104 (0.806–1.513)	-	
Management-related factors	11.7 (9.9–13.8)	10.8 (9.1–12.8)	12.6 (10.6–14.9)	<0.001	0.844 (0.793–0.898)	0.005	0.907 (0.848–0.971)
Unsolvable queen problems	3.8 (3.1–4.6)	3.9 (3.2–4.8)	3.6 (3.0–4.4)	0.143	1.081 (0.974–1.201)	-	
Dead colonies (mortality)	7.8 (5.9–10.2)	6.8 (5.1–9.0)	8.9 (6.7–11.6)	<0.001	0.753 (0.699–0.811)	<0.001	0.802 (0.740–0.869)
Empty hives (CDS)	3.7 (2.0–7.0)	2.9 (1.5–5.6)	4.7 (2.5–8.8)	<0.001	0.611 (0.554–0.675)	<0.001	0.589 (0.527–0.657)
No food (starvation)	1.4 (0.7–2.7)	1.7 (0.8–3.2)	1.1 (0.6–2.2)	<0.001	1.493 (1.251–1.781)	<0.001	1.685 (1.392–2.040)

^a^ Controlled for the year of the study (random effect); ^b^ controlled for the year of the study and geographical region (random effects), as well as the number of colonies owned by a beekeeper, percentage of replaced queens, practice of migration of colonies, varroosis monitoring, and varroosis treatment (fixed effects).

## Data Availability

Data available from the authors on request.

## Supplementary Materials

The following supporting information can be downloaded at: https://www.mdpi.com/article/10.3390/insects13121128/s1. Figure S1: Polish version of COLOSS questionnaire; Table S1: The multivariable analysis of the relationship between the use of screened bottom boards and the overall colony loss rate controlled for potential confounders; Table S2: The multivariable analysis of the relationship between the use of screened bottom boards and the management-related colony loss rate controlled for potential confounders; Table S3: The multivariable analysis of the relationship between the use of screened bottom boards and the management-related colony loss rate due to dead colonies (mortality rate) controlled for potential confounders; Table S4: The multivariable analysis of the relationship between the use of screened bottom boards and the management-related colony loss rate due to dead colonies (mortality rate) in which empty hives were observed (CDS), controlled for potential confounders; Table S5: The multivariable analysis of the relationship between the use of screened bottom boards and the management-related colony loss rate due to dead colonies (mortality rate) in which a lack of food was observed (starvation), controlled for potential confounders.
