# Supplementary material for: Two Faces of the Screened Bottom Boards—An Ambiguous Influence on the Honey Bee Winter Colony Loss Rate
Source: Insects. 2022 Dec 7;13(12):1128. doi: 10.3390/insects13121128 (PMC9784936; doi:10.3390/insects13121128)
Supplement: Supplementary file 1 [file insects-13-01128-s001.zip › insects-2057611-supplementary.pdf]

Supplementary files

COLOSS – honey bee colony loss and survival survey 2019/2020

Please send the questionnaire survey to: Laboratory of Bee Diseases, Department of Pathology and Veterinary Diagnostics, Warsaw University of Life Sciences-SGGW, 02-786 Warszawa, ul Ciszewskiego 8
or to e-mail adress: ewa_mazur@sggw.pl

until 17th June 2020 r.
Phone number : 22 59 361 40

Wydział Medycyny Weterynaryjnej SGGW

Postal code of residence and region____________________________
ID number from last survey or identification password____________________
e-mail____________________________________

❶ To describe the location of your main apiary or operation, please state:
a) The name of city/town/village near to you apiary____________________
b) The postal code of the apiary and the name of the region ____________

❷ How many apiaries do you have? [ ]

❸ If you have more than one apiary, are all your apiaries within a distance of about 15 km of each other? If you have only one apiary, please answer yes.
a) yes **b) no** **c) don't know**

E① ***Number of colonies, losses and status of the colony post-winter. Please consider winter as a period between the moment that you finished the pre-winter preparations for your colonies and the start of the new foraging season. In this questionnaire we try to gather information about production colonies. Please consider colonies which are queenright and strong enough to provide a honey harvest as production colonies.***

❹ How many production colonies did you have before winter 2019-2020? [ ]

E② ***In the next questions you are asked for numbers of colonies lost. Please consider a colony as lost if is dead (or reduced to a few hundred bees), OR lost due to natural disaster, OR ALIVE but with queen problems, like drone laying queens or no queen at all, which you couldn't solve. EACH LOST COLONY SHOULD BE INCLUDED IN ONLY ONE OF THESE THREE CATEGORIES.***

❺ How many of these wintered colonies (❹) did you lose, because they were ALIVE but had unsolvable queen problems. If none, please answer 0. [ ]
❻ How many of these wintered colonies (❹) did you lose, because of natural disaster (fire, flood, bear, vandalism...)? [ ]

❼ How many of these wintered colonies (❹) did you lose, because they were dead (including those that have „disappeared")? [ ]
❽ How many of the DEAD colonies (❼) ...
a) ...had no or only a few dead bees in or in front of the empty hive? [ ]
b) ...had dead workers in cells and no food present in the hive (sign of starvation)? [ ]

❾ How many of the wintered colonies (❹) were weak, but queenright after winter 2019-2020? [ ]

E③ ***We would like to calcite increases and decreases in the number of colonies, so if you had colonies in spring 2019 and remember how many you had, please answer the following two questions, considering spring as the start of the foraging season:***

❿ How many production colonies did you have: a) spring 2019 (last year)? [ ]
b) spring 2020 ? [ ]

E④ ***Conditions in the colonies, the environment around the apiary, and the management:***

⓫ How many of the wintered colonies has a new queen in 2019? [Don't know] [ ]

⓬ To what extent did you observe queen problems in your colonies during the foraging season of 2019 compared to what you usually have: **a) more** **b) normal** **c) less** **d) don't know**

⓭ Compared with your colonies with old Queen, have your colonies with young queens survived winter:
a) better **b) no differently** **c) worse** **d) don't know**

⓮ How many of your surviving colonies has a large amount of faeces inside the hive after winter? [ ]

Wydział Medycyny Weterynaryjnej SGGW

⓯ Did you migrate any of your colonies at least once for honey production or pollination in 2019?

a) yes **b) no** **c) don't know**

⓰ Approximately what proportion of brood combs did you replace with comb foundation per colony in 2019?

a) 0% **b) 1-30%** **c) 31-50%** **d) more than 50%**

⓱ Did the majority of your bee colonies have a significant flow on one or more of the following plants in 2019?

orchards	**a) yes**	**b) no**	**c) don't know**
oil seed rape	**a) yes**	**b) no**	**c) don't know**
maize	**a) yes**	**b) no**	**c) don't know**
heather	**a) yes**	**b) no**	**c) don't know**
autumn forage crops	**a) yes**	**b) no**	**c) don't know**
honeydew	**a) yes**	**b) no**	**c) don't know**

⓲ Have you monitored your colonies for Varroa during the period April 2019 – March 2020?

a) yes **b) no** **c) don't know**

⓳ Have you treated your colonies against Varroa during the period April 2019 – March 2020?

a) yes **b) no** **c) don't know**

⓴Could you please indicate the months when you monitored your production colonies for Varroa AND also indicate when you STARTED a Varroa treatment or management plan during the period April 2019 – March 2020?	Month in which each measure started:											
	2019									2020		
**Method/Period**	April	May	June	July	August	September	October	November	December	January	February	March
Monitoring of Varroa infestation level												
Drone brood removal												
Hyperthermia (heat treatment of brood/bees)												
Other biotechnical method (e.g. trapping comb, complete brood removal, queen confinement)												
Formic acid – short term												
Formic acid – long term												
Oxalic acid – trickling (Oxybee, Varromed)												
Oxalic acid – sublimation (evaporation)												
Thymol (e.g. Apiguard, ApilifeVar, Thymovar)												
Flumethrin (Bayvarol, PolyVar Yellow)												
Amitraz in strips (Biowar, Apivar)												
How many strips did you use and how long did they stay in the hive?												
Amitraz – fumigation (Apiwarol)												
How many times and at what intervals did you apply fumigation with amitraz?												
Another chemical product (……………………………………………)												
Another method (……………………………………………………………)												

21 What particular measures apply for the majority of your beekeeping:

a)	Screened bottom board in winter	**a) yes**	**b) no**	**c) don't know**
b)	Insulated hives in winter	**a) yes**	**b) no**	**c) don't know**
c)	Hives made from synthetic materials	**a) yes**	**b) no**	**c) don't know**
d)	Certified organic beekeeping	**a) yes**	**b) no**	**c) don't know**
e)	Queens bred from Varroa-tolerant stock	**a) yes**	**b) no**	**c) don't know**
f)	Small brood cell size (5.1 mm or less)	**a) yes**	**b) no**	**c) don't know**
g)	Comb without foundation	**a) yes**	**b) no**	**c) don't know**
h)	Purchase wax from outside own operation	**a) yes**	**b) no**	**c) don't know**

22 If there were symptoms in the apiary during the 2019 season that raised the suspicion of acute bee poisoning (sudden appearance of large numbers of dead or paralyzed bees in the front of the hives), in what month(s) was it? Don't know

Figure S1. The Polish version of the COLOSS questionnaire.

Table S1. The multivariable analysis of the relationship between the use of screened bottom boards and the overall colony loss rate controlled for potential confounders.

**Variable**	**Regression coefficient (standard error)**	**Parameter statistics**	***p*-value**	Adjusted odds ratio **(CI 95%)**
		Fixed effects		
Intercept	−1.514 (0.148)	-	-	-
Screened bottom board				
No	Reference category			
Yes	−0.100 (0.034)	−2.963	0.003*	0.905 (0.847 – 0.967)
Number of colonies	−0.001 (0.001)	−7.585	<0.001*	0.999 (0.999 – 0.999)
Migration of colonies				
No	Reference category			
Yes	−0.098 (0.035)	−2.781	0.006*	0.906 (0.846 – 0.971)
Proportion of queens replaced	−0.295 (0.078)	−3.804	<0.001*	0.744 (0.639 – 0.867)
Varroosis monitoring				
No	Reference category			
Yes	−0.044 (0.039)	−1.135	0.257	0.957 (0.887 – 1.032)
Varroosis treatment				
No	Reference category			
Yes	−0.134 (0.085)	−1.584	0.114	0.874 (0.740 – 1.033)
		Random effects		
Year of the study	0.078 (0.030)	2.580	0.010*	-
Region in which the apiary located	0.026 (0.027)	0.975	0.329	-

* significant at the significance level of 0.05.

Table S2. The multivariable analysis of the relationship between the use of screened bottom boards and the management-related colony loss rate controlled for potential confounders.

**Variable**	**Regression coefficient (standard error)**	**Parameter statistics**	***p*-value**	Adjusted odds ratio **(CI 95%)**
		Fixed effects		
Intercept	−1.477 (0.150)	-	-	-
Screened bottom board				
No	Reference category			
Yes	−0.097 (0.034)	−2.833	0.005*	0.907 (0.848 – 0.971)
Number of colonies	−0.001 (0.001)	−7.35	<0.001*	0.999 (0.999 – 0.999)
Migration of colonies				
No	Reference category			
Yes	−0.074 (0.036)	−2.067	0.039*	0.928 (0.865 – 0.996)
Proportion of queens replaced	−0.426 (0.079)	−5.381	<0.001*	0.653 (0.559 – 0.763)
Varroosis monitoring				
No	Reference category			
Yes	−0.051 (0.039)	−1.293	0.196	0.951 (0.88 – 1.027)
Varroosis treatment				
No	Reference category			
Yes	−0.166 (0.085)	−1.946	0.052	0.847 (0.717 – 1.001)
		Random effects		
Year of the study	0.028 (0.028)	0.976	0.329	-
Region in which the apiary located	0.078 (0.030)	2.581	0.010*	-

* significant at the significance level of 0.05.

Table S3. The multivariable analysis of the relationship between the use of screened bottom boards and the management-related colony loss rate due to dead colonies (mortality rate) controlled for potential confounders.

Variable	Regression coefficient (standard error)	Parameter statistics	*p*-value	Adjusted odds ratio (CI 95%)
Fixed effects				
Intercept	−2.102 (0.206)	-	-	-
Screened bottom board				
No	Reference category	-	-	-
Yes	−0.220 (0.041)	−5.377	<0.001*	0.802 (0.740 – 0.869)
Number of colonies	−0.001 (0.001)	−8.786	<0.001*	0.999 (0.999 – 0.999)
Migration of colonies				
No	Reference category	-	-	-
Yes	0.053 (0.043)	1.245	0.213	1.055 (0.970 – 1.147)
Proportion of queens replaced	−0.349 (0.094)	−3.694	<0.001*	0.706 (0.586 – 0.849)
Varroosis monitoring				
No	Reference category	-	-	-
Yes	−0.102 (0.046)	−2.223	0.026*	0.903 (0.825 – 0.988)
Varroosis treatment				
No	Reference category	-	-	-
Yes	0.050 (0.105)	0.478	0.633	1.051 (0.856 – 1.291)
Random effects				
Year of the study	0.073 (0.074)	0.986	0.324	-
Region in which the apiary located	0.090 (0.035)	2.569	0.010*	-

* significant at the significance level of 0.05.

Table S4. The multivariable analysis of the relationship between the use of screened bottom boards and the management-related colony loss rate due to dead colonies (mortality rate) in which the empty hives were observed (Colony Depopulation Syndrome, CDS) controlled for potential confounders.

Variable	Regression coefficient (standard error)	Parameter statistics	*p*-value	Adjusted odds ratio (CI 95%)
Fixed effects				
Intercept	-2.826 (0.401)			
Screened bottom board				
No	Reference category	-	-	-
Yes	−0.530 (0.056)	−9.453	<0.001*	0.589 (0.527 – 0.657)
Number of colonies	−0.001 (0.001)	−6.764	<0.001*	0.999 (0.998 – 0.999)
Migration of colonies				
No	Reference category	-	-	-
Yes	0.213 (0.059)	3.637	<0.001*	1.237 (1.103 – 1.388)
Proportion of queens replaced	−0.412 (0.124)	−3.312	<0.001*	0.663 (0.519 – 0.846)
Varroosis monitoring				
No	Reference category	-	-	-
Yes	−0.190 (0.062)	−3.072	0.002*	0.827 (0.732 – 0.934)
Varroosis treatment				
No	Reference category	-	-	-
Yes	−0.005 (0.140)	−0.034	0.973	0.995 (0.757 – 1.309)
Random effects				
Year of the study	0.360 (0.363)	0.994	0.320	-
Region in which the apiary located	0.303 (0.121)	2.516	0.012*	-

* significant at the significance level of 0.05.

Table S5. The multivariable analysis of the relationship between the use of screened bottom boards and the management-related colony loss rate due to dead colonies (mortality rate) in which the lack of food was observed (starvation) controlled for potential confounders.

Variable	Regression coefficient (standard error)	Parameter statistics	*p*-value	Adjusted odds ratio (CI 95%)
Fixed effects				
Intercept	−3.086 (0.392)	-	-	-
Screened bottom board				
No	Reference category	-	-	-
Yes	0.522 (0.097)	5.364	<0.001*	1.685 (1.392 – 2.040)
Number of colonies	−0.002 (0.001)	−4.699	<0.001*	0.998 (0.998 – 0.999)
Migration of colonies				
No	Reference category	-	-	-
Yes	0.211 (0.100)	1.984	0.034*	1.235 (1.016 – 1.501)
Proportion of queens replaced	−1.459 (0.229)	−6.376	<0.001*	0.232 (0.148 – 0.364)
Varroosis monitoring				
No	Reference category	-	-	-
Yes	−0.392 (0.101)	−3.887	<0.001*	0.676 (0.555 – 0.824)
Varroosis treatment				
No	Reference category	-	-	-
Yes	−0.541 (0.178)	−3.045	0.002*	0.582 (0.411 – 0.825)
Random effects				
Year of the study	0.319 (0.326)	0.980	0.327	-
Region in which the apiary located	0.130 (0.062)	2.101	0.036*	-

* significant at the significance level of 0.05.
